# Spatially and Temporally Resolved Ambient PM_2.5_ in Relation to Preterm Birth

**DOI:** 10.3390/toxics9120352

**Published:** 2021-12-14

**Authors:** Whitney Cowell, Elena Colicino, Xueying Zhang, Rachel Ledyard, Heather H. Burris, Michele R. Hacker, Itai Kloog, Allan Just, Robert O. Wright, Rosalind J. Wright

**Affiliations:** 1 Department of Environmental Medicine and Public Health, Icahn School of Medicine at Mount Sinai, New York, NY 10128, USA; elena.colicino@mssm.edu (E.C.); xueying.zhang@mssm.edu (X.Z.); ikloog@bgu.ac.il (I.K.); allan.just@mssm.edu (A.J.); robert.wright@mssm.edu (R.O.W.); rosalind.wright@mssm.edu (R.J.W.); 2 Division of Neonatology, Children’s Hospital of Philadelphia, Philadelphia, PA 19104, USA; ledyardr@chop.edu (R.L.); burrish@chop.edu (H.H.B.); 3 Department of Pediatrics, Perelman School of Medicine, University of Pennsylvania, Philadelphia, PA 19104, USA; 4 Obstetrics and Gynecology, Beth Israel Deaconess Medical Center, Harvard Medical School, Boston, MA 02215, USA; mhacker@bidmc.harvard.edu; 5 Department of Geography and Environmental Development, Ben-Gurion University of the Negev, Beersheba 8410501, Israel; 6 Institute for Exposomic Research, Icahn School of Medicine at Mount Sinai, New York, NY 10029, USA; 7 Department of Pediatrics, Kravis Children’s Hospital, Icahn School of Medicine at Mount Sinai, New York, NY 10029, USA

**Keywords:** fine particulate matter, PM_2.5_, pregnancy, prenatal, preterm birth, fetal sex, air pollution

## Abstract

Growing evidence suggests that maternal exposure to ambient fine particulate matter (PM_2.5_) during pregnancy is associated with preterm birth; however, few studies have examined critical windows of exposure, which can help elucidate underlying biologic mechanisms and inform public health messaging for limiting exposure. Participants included 891 mother–newborn pairs enrolled in a U.S.-based pregnancy cohort study. Daily residential PM_2.5_ concentrations at a 1 × 1 km^2^ resolution were estimated using a satellite-based hybrid model. Gestational age at birth was abstracted from electronic medical records and preterm birth (PTB) was defined as <37 completed weeks of gestation. We used Critical Window Variable Selection to examine weekly PM_2.5_ exposure in relation to the odds of PTB and examined sex-specific associations using stratified models. The mean ± standard deviation PM_2.5_ level averaged across pregnancy was 8.13 ± 1.10 µg/m^3^. PM_2.5_ exposure was not associated with an increased odds of PTB during any gestational week. In sex-stratified models, we observed a marginal increase in the odds of PTB with exposure occurring during gestational week 16 among female infants only. This study does not provide strong evidence supporting an association between weekly exposure to PM_2.5_ and preterm birth.

## 1. Introduction

In the United States, approximately 10% of newborns are born preterm, defined as birth before 37 weeks of gestation [1]. Preterm birth (PTB) is associated with an increased risk of infant mortality and a range of morbidities that can persist into adulthood with potential lifelong consequences for health and wellbeing [2,3,4,5]. While the risk factors for PTB remain incompletely understood, a growing body of research has linked maternal exposure to fine ambient particulate matter (PM_2.5_) during pregnancy with this adverse pregnancy outcome [6,7].

A notable feature of most prior studies examining PM_2.5_ in relation to birth outcomes is a focus on average exposure across the course of pregnancy, which fails to account for the time-varying nature of ambient air pollution exposure and does not allow for more discrete, etiological windows of susceptibility to be identified. Understanding these critical windows can guide the implementation of additional protections for the fetus during specific gestational periods and could help elucidate the biological mechanisms underlying the pathophysiology of adverse health outcomes, such as PTB [8,9]. Furthermore, most prior studies, including the few with temporally resolved exposure estimates, have relied on birth registry data for ascertaining information on gestational length [10,11,12]. While this design allows for a large sample to be aggregated, information on key covariates can be limited and exposure estimates are often cursory at the census tract or zip code level. Many prior studies have also relied on sparse networks of ground monitors to derive PM_2.5_ estimates, which can lead to exposure misclassification, as well as selection bias if the sample of participants residing near monitors is not representative of the population. Finally, many prior studies have examined associations between PM_2.5_ and PTB in the setting of relatively high exposure levels, which may not generalize to lower-level (i.e., below the U.S. EPA annual standard of 12 µg/m^3^) exposure that is typical across the United States [13,14]. Understanding whether adverse health effects extend to lower exposure ranges is important for evaluating whether current air quality standards are sufficient for protecting the public’s health.

In the present study, we addressed several of the above noted limitations by examining weekly exposure to lower-level PM_2.5_, estimated at a 1 × 1 km^2^ spatial resolution, in relation to PTB using data from a prospective, ethnically diverse pre-birth cohort based in the northeastern United States. We also explored differences by spontaneous versus iatrogenic phenotypes of PTB, which are etiologically distinct. Finally, because the incidence of PTB varies by fetal sex [15] and prior research has documented sex-specific associations between PM_2.5_ and birth outcomes [16], we examined differences by infant sex.

## 2. Methods

### 2.1. Study Sample

Participants included mother-newborn pairs enrolled in the PRogramming of Intergenerational Stress Mechanisms (PRISM) pregnancy cohort, which recruited from prenatal clinics in Boston from 2011–2013 and New York City from 2013-present. Participants were ineligible if they were younger than 18 years of age, HIV positive, pregnant with multiples, non-English or -Spanish speaking, or if they drank more than seven alcoholic drinks per week before pregnancy or any alcohol after pregnancy recognition. At the time of this analysis, 1119 eligible, enrolled participants had delivered a live-born infant with no major congenital anomalies noted during pregnancy or at birth that would impede continued participation in the study. Of these participants, we excluded 137 whose addresses had not yet been successfully geocoded and 78 with incomplete covariate data. We further restricted the sample to births occurring after 32 weeks of gestation to allow for equal exposure timing, resulting in a final analytic sample of 891 participants (Boston: *n* = 375, New York City: *n* = 516; Supplemental Figure S1). Women included in the analytic dataset were more likely to be white, non-Hispanic, to have more than a high school education and to be non-smoke exposed compared to participants enrolled in the cohort, but excluded from the analytic sample (Supplemental Table S1). These differences reflect the fact that participants from the Boston study site were enrolled earlier and were more likely to have geocoded address data available at the time of this analysis. In turn, participants enrolled from Boston were more likely to be white, non-Hispanic, more highly educated and non-smoke exposed compared to participants enrolled from New York City. Written informed consent was obtained from women prior to study participation in their preferred language. All study procedures were approved by the Institutional Review Boards at the Brigham and Women’s Hospital in Boston and the Icahn School of Medicine at Mount Sinai in New York City.

### 2.2. Fine Particulate Matter Exposure

We geocoded maternal residential address during pregnancy, accounting for any residential moves, using ArcGIS software (Redlands, CA, USA) as previously described [17]. For each participant, we estimated PM_2.5_ exposure for each day of pregnancy using an adaptation of a previously described satellite-based hybrid model (18). Inputs included Aerosol Optical Depth (AOD) products from the two Moderate-Resolution Imaging Spectroradiometer (MODIS) instruments on the NASA Terra and Aqua satellites, in combination with PM_2.5_ monitoring data and a series of spatiotemporal predictors (height of the planetary boundary layer, percentage of developed area, air temperature, relative humidity, and others) [18]. Using these inputs, we applied an extreme gradient boosting (XGBoost) modeling approach to predict daily, residential PM_2.5_ and implemented a recursive feature selection process to arrive at a parsimonious model. The model demonstrates excellent predictions of withheld observations (RMSE of 2.10 µg/m^3^ and RMSE of 3.11 µg/m^3^ in our spatial cross-validation). Similar to PM_2.5_ AOD products, daily land surface temperature was obtained from the MODIS instruments [19]. These measures were calibrated to the ambient air temperature at the reference height (2 m above ground) using ground monitoring data derived from the National Climate Data Center, the Meteorological Assimilation Data Ingest System of the National Oceanic and Atmospheric Administration, and a large number of aggregated nongovernmental meteorologic stations. This calibration also included a temporal smoothing algorithm to account for location, season, year, land-use regression terms for greenness, elevation, and land use. Model performance was assessed following the approach described for PM_2.5_ estimates. Daily measures of both PM_2.5_ and temperature were measured at a 1 × 1 km^2^ or higher spatial resolution.

### 2.3. Gestational Age at Birth

For the majority of participants (95.7%), we determined gestational age at delivery based on best obstetric estimate ascertained from review of electronic medical records. This is derived from first-trimester ultrasound revision or confirmation of last menstrual period dating and is determined by the participant’s obstetrician. If no obstetric estimate was available, we calculated gestational age using date of delivery and maternal-reported last menstrual period (4%) or relied on maternal self-report (0.3%). According to American College of Obstetricians and Gynecologist guidelines, we defined PTB as birth before 37 completed weeks of gestation. We additionally categorized PTB into spontaneous (sPTB, for example, preterm labor, spontaneous rupture of membranes) or iatrogenic (iPTB, for example, clinician-initiated due to a maternal or fetal health condition, such as preeclampsia or intrauterine growth restriction) as previously described using a standardized protocol [20].

### 2.4. Covariates

Information on maternal age, race/ethnicity, highest level of education, and parity was ascertained by questionnaire during a structured interview conducted during pregnancy. Information on smoking and exposure to environmental tobacco smoke (ETS) was assessed during pregnancy and again during the immediate post-partum period. Women were considered smoke exposed if they reported ever smoking during pregnancy or exposure to ETS for one hour or more per week during pregnancy. To minimize biases in estimates of association, we identified potential confounders using directed acyclic graph (DAG) analysis (Supplemental Figure S2) [21]. We based the conditional dependencies defined by our DAG on review of previous literature and knowledge of factors influencing PTB and/or PM_2.5_ exposure. We adjusted for education, used as an indicator of socioeconomic position, race/ethnicity, which has been linked to PTB prevalence and may be a factor determining an individual’s residential neighborhood and thus propensity for PM_2.5_ exposure, and surface temperature, which is a predictor of seasonal trends in PM_2.5_ and may be independently associated with PTB [22]. We additionally adjusted for maternal age, parity, and cigarette smoke exposure, which we considered important precision variables linked with the risk of PTB; adjusting for these factors did not open any backdoor paths as defined by our DAG.

### 2.5. Statistical Analysis

**Figure 1:**
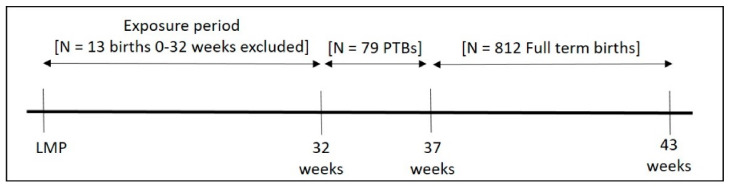
Schematic outlining exposure and outcome timing.

We examined descriptive statistics for the PM_2.5_ data, gestational age data, and each covariate, and then visually inspected the distributions of each variable using histograms and boxplots. We performed Critical Window Variable Selection (CWVS) to examine weekly PM_2.5_ exposure during pregnancy in relation to the odds of PTB. CWVS is a recently developed Bayesian variable selection method for identifying critical windows of susceptibility to a time-varying exposure. Briefly, during the selection and estimation process, temporal smoothness is introduced using a flexible cross-covariance model based on the linear model of coregionalization [23]. CWVS avoids the over smoothing that often occurs with the use of Gaussian processes and has been shown to perform well with exposure data that have a high temporal correlation, including in simulation studies of air pollution and PTB [23]. We constructed weekly PM_2.5_ exposure matrices that spanned the period between the mother’s LMP and 32 weeks of gestation. Because CWVS requires an equal exposure period for all participants and to avoid bias introduced from participants who would leave the risk set, we excluded 13 women who delivered before 32 weeks [10], as illustrated by the schematic provided in Figure 1. We examined intercept-only models and models adjusted for maternal age, parity, race/ethnicity, education, cigarette smoke exposure during pregnancy, and mean temperature during the first 32 gestational weeks. Continuous covariates were centered and standardized to have a mean of zero and standard deviation of one. We fit each model using 10,000 Markov Chain Monte-Carlo iterations, discarding the first 1000 as a burn in period, and assessed convergence through visual inspection of trace plots. We considered sex differences using stratified models. In all models, effect estimates are interpreted as the change in the odds of PTB for an interquartile range (IQR) increase in PM_2.5_ exposure. We ran a parallel set of exploratory models to examine PM_2.5_ in relation to the odds of sPTB or iPTB. In models examining sPTB, iPTBs were excluded and in models examining iPTBs, sPTBs were excluded (i.e., they did not contribute to the reference group). In both models, 11 women missing information on PTB phenotype were excluded. We did not consider sex differences in models examining PTB phenotypes due to sample size limitations. We also explored PTB models excluding participants diagnosed with gestational hypertension, pre-eclampsia/eclampsia, or gestational diabetes during pregnancy (*n* = 138), as well as those missing information on these pregnancy-related complications (*n* = 45). Finally, in two separate supplemental analyses (Supplementary Materials), we examined our main CWVS models additionally adjusting for (1) season of maternal LMP (spring: March-May, summer: June-August, fall: September-November, winter: December-February), (2) year of birth (2011–2019), or (3) study site (Boston vs. New York City). All statistical analyses were performed in R v3.6.2; CWVS was performed using the *CWVS* R package, which can be accessed through GitHub (https://github.com/warrenjl/CWVS) (accessed on 12 May 2021) [23].

## 3. Results

Table 1 provides sociodemographic characteristics for the study sample. On average, women were 29 years old at enrollment and the majority self-identified as Black/Black-Hispanic (43.2%) or white-Hispanic (35.6%), with the remainder identifying as white, non-Hispanic (16.5%) or other race/ethnicity (4.7%). Approximately 20% of women had less than a high school education, 66% were nulliparous and 11% reported exposure to cigarette smoke during pregnancy. Average PM_2.5_ exposure across the first 32 weeks of pregnancy was approximately normally distributed with a mean ± standard deviation (SD) of 8.13 ± 1.10 µg/m^3^ and interquartile range (IQR) of 1.56 µg/m^3^. PM_2.5_ exposure did not significantly vary between mothers who delivered preterm versus term (Supplemental Figure S3). The mean ± SD for temperature was 12.10 ± 4.31C and the IQR was 7.74C. The sample included 79 (8.9%) infants born preterm, which is slightly lower than the U.S. incidence of 10%. Information on PTB subtype was not available for 11 participants; of the remaining 68 PTBs, 33 (48.5%) were spontaneous and 35 (51.5%) were iatrogenic in nature.

**Figure 2:**
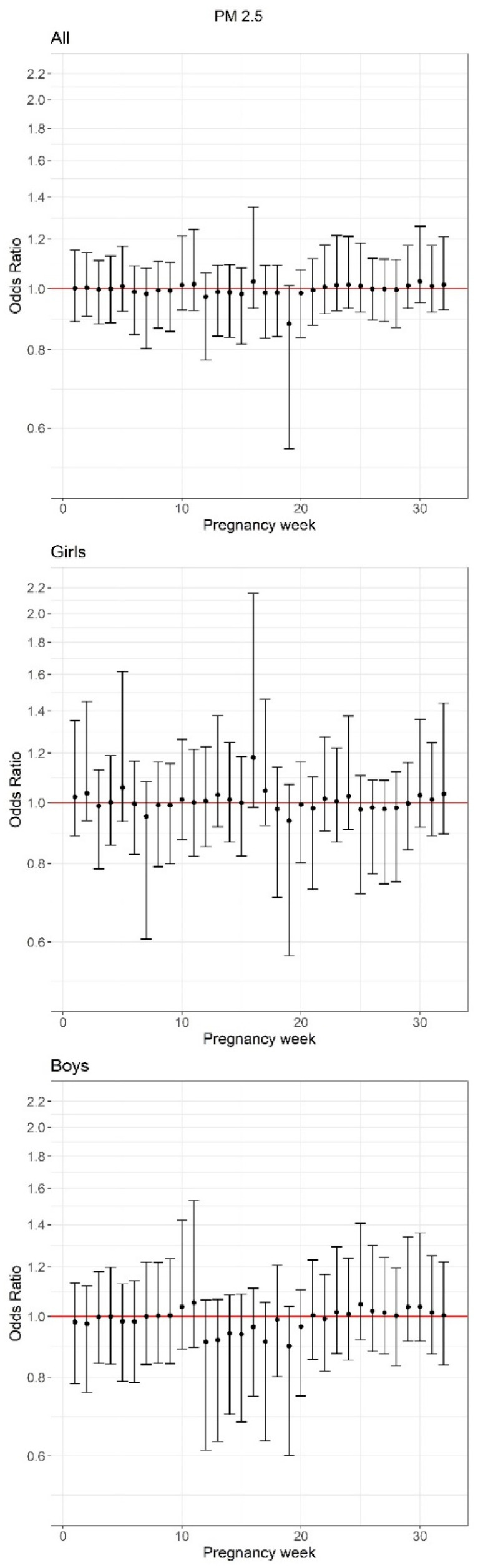
Posterior mean and 95% credible intervals from overall and sex-stratified adjusted Critical Window Variable Selection models examining an interquartile range increase in weekly PM_2.5_ in relation to the odds of preterm birth.

We detected no statistically significant associations between weekly exposure to PM_2.5_ and PTB when considering the sample overall; however, we observed a marginal decrease in the odds of PTB with exposure occurring during week 19 (Figure 2). The results of intercept-only models did not substantially vary from models adjusted for covariates (Supplemental Figure S4). In adjusted sex-stratified models, we observed a marginal increase in the odds of PTB with exposure occurring during week 16 of gestation among female newborns only (Figure 2). Similar to main analyses, in exploratory models considering PTB phenotypes, we did not detect any significant associations with weekly PM_2.5_ exposure. The marginal protective association at week 19 was apparent only among the subset of iPTBs (Supplemental Figure S5). Likewise, results from models excluding participants with gestational hypertension, gestational diabetes, or pre-eclampsia/eclampsia, were similar to main results, with no statistically significant associations detected (Supplemental Figure S6). Finally, supplemental models additionally adjusting for season of maternal LMP (Supplemental Figure S7), child year of birth (Supplemental Figure S8), or study enrollment site (Supplemental Figure S9) were not meaningfully different from the results of primary analyses presented in Figure 2.

## 4. Discussion

Several studies have examined trimester-specific windows of exposure to PM_2.5_ in relation to PTB with inconsistent results [24,25,26,27,28,29]. Notably, this design may be insufficient to identify susceptible windows if the relevant biological responses do not align with clinically defined trimesters. Our group has recently demonstrated that trimester-specific models produce biased estimates and may identify inaccurate windows [8]. Exposure levels may also be sensitive to the temporal scale of aggregation. For example, a recent study demonstrated that PM_2.5_ averaged across pregnancy weeks 1–12 versus weeks 3–8 resulted in exposure reclassification by at least one quartile for 37% of the sample [30]. A few studies have alternatively employed time-series approaches to examine monthly or weekly periods of exposure in relation to PTB. Similar to trimester-averaged exposure models, these studies have reported inconsistent findings and no clear etiologically relevant period of susceptibility has been identified. For example, studies have reported susceptible windows during both early and late gestation [31], early gestation only [32], middle gestation only [11], late gestation only [33], early and middle gestation [10], middle and late gestation [34], or have failed to identify any susceptible window [35,36]. A notable limitation of prior studies with high temporal resolution is that spatial resolution has been limited, with PM_2.5_ exposure typically estimated at the zip code or county level using ground monitoring data. This can result in exposure misclassification or selection bias if participants included due to proximity to monitors are not representative of the sample overall. In the present study, we addressed these limitations by estimating daily PM_2.5_ exposure at a 1 × 1 km^2^ resolution. We additionally applied the recently developed CWVS method to identify critical windows of susceptibility, which accounts for the correlation in exposure across pregnancy and has been shown in simulation studies to be less susceptible to over smoothing during estimation of risk parameters compared to other time series methods [12,23]. Using this approach, we did not detect an association between PM_2.5_ exposure during any gestational week and PTB. Although we had high spatial and temporal resolution, it is notable that our sample size was small and exposure levels were relatively low with somewhat limited variability, which together may have reduced our ability to detect an association. We were also limited to assessing ambient exposure at the mother’s residential address, which does not take into account time-activity patterns or exposure to particulate air pollution in the indoor environment.

Unexpectedly, we found a marginal decrease in the odds of PTB with PM_2.5_ exposure during gestational week 19, which remained only among the subset of iPTBs. A few prior studies have documented similar inverse associations [24,37]; however, the reason underlying this directionality is unclear. One prior study found that an inverse association between PM_2.5_ and PTB was reversed when multipollutant models that included criteria and traffic-related air pollutants were considered, suggesting that the effects of PM_2.5_ may in part depend on the mixture of joint exposures it occurs with [38,39]. Why week 19 exposure appeared somewhat protective only among iPTBs, which include pregnancies complicated by gestational diabetes, gestational hypertension and pre-eclampsia, also remains unknown.

We did not identify meaningfully different associations by newborn sex, with the exception that among females only there was a marginal positive association with PM_2.5_ exposure at gestational week 16 (early 2nd trimester). This finding is consistent with recent murine research that demonstrated pregnant mice exposed to concentrated air particles (150 µg/m^3^) during the late 1st trimester and 2nd trimester displayed significantly reduced gestational length; however, sex differences were not considered in that study [40]. The placenta plays key roles in pregnancy maintenance and the onset of parturition. Mechanistic research supports that PM_2.5_ affects several placental parameters, including weight, vasculature, perfusion, and other indicators of functional morphology [41]. Recent in vitro work has also shown that acute exposure of 1st trimester trophoblast cells to PM_2.5_ results in decreased production of human chorionic gonadotropin (hCG), which is critical for placental development and progression of a healthy pregnancy, as well as increased production of the pro-inflammatory cytokine interleukin-6 (IL-6), among other changes [42]. This imbalance between downregulated reproductive hormones and stimulated inflammation could set in motion a cascade of molecular changes ending in preterm delivery [43]. Notably, a recent study of 610 pregnancies, found that increased IL-6 and c-reactive protein between gestational weeks 12–20 was associated with decreased gestational age and an increased odds of preterm delivery [44]. Unfortunately, the study did not consider sex-specificity; however, placental cytokine levels and responses have been shown to vary by fetal sex [45]. Alternatively, we acknowledge our finding of a marginally increased odds of PTB among females could have been spurious given our limited sample size.

Overall, the inconsistencies across studies, including those examining weekly or monthly exposure, may be attributable to several factors, including differences in study designs, PM_2.5_ exposure modeling, PM_2.5_ level and chemical composition, and demographic and lifestyle characteristics of different samples. This heterogeneity makes it difficult to identify repeatable results and draw conclusions about susceptible windows of exposure. Strengths of the present study include the diversity of the sample and rigorous characterization of PTB, including iatrogenic and spontaneous phenotypes, based on review of medical records. We estimated exposure levels on a daily basis, which allowed us to investigate potential critical windows of exposure using the recently developed CWVS approach. We also estimated PM_2.5_ and temperature at a 1 × 1 km^2^ grid using a robust satellite-based hybrid model with bias correction. However, despite the high spatial and temporal resolution, our estimates do not fully capture an individual’s immediate exposure as a personal sampling device or biological marker would. As a consequence, our estimates are susceptible to potential exposure misclassification. As previously noted, we also did not have data on time-activity patterns, including information about time spent outdoors, which could plausibly be non-differential by PTB status if risk factors for PTB (e.g., hypertension, diabetes) relate to physical activity. Additionally, although individuals are exposed to mixtures of ambient air pollutants in the environment, we were limited to investigating only PM_2.5_ in this study. Future research examining ambient air pollution mixtures will more accurately reflect true exposures in the community and will advance our understanding of how co-exposures interact to affect health. Finally, while our prospective birth cohort design allowed us to examine critical windows and control for individual-level covariates, our sample size was limited. 

In sum, we did not detect a critical window of PM_2.5_ exposure for the risk of PTB. Future research with a large sample size in combination with highly spatially and temporally resolved PM_2.5_ estimates may help to further elucidate gestational windows of susceptibility to PM_2.5_.

## Figures and Tables

**Table 1:** Participant characteristics by preterm (<37 weeks of gestation) and term (≥37 weeks) birth status. Values are mean ± SD or *n* (%).

	All (*n* = 891, 100%)	Preterm (*n* = 79, 8.9%)	Term (*n* = 812, 91.1%)
Maternal age	29.1 ± 5.8	30.4 ± 6.0	28.9 ± 5.8
Race/ethnicity			
White, non-Hispanic	147 (16.5)	9 (6.1)	138 (93.9)
White-Hispanic	317 (35.6)	33 (10.4)	284 (89.6)
Black/Black-Hispanic	385 (43.2)	35 (9.1)	350 (90.9)
Other	42 (4.7)	2 (4.8)	40 (95.2)
Education			
<High school	183 (20.5)	20 (10.9)	163 (89.1)
High school degree	444 (49.8)	39 (8.8)	405 (91.2)
>High school	264 (29.6)	20 (7.6)	244 (92.4)
Parity			
Nulliparous	303 (34.0)	16 (5.3)	287 (94.7)
Multiparous	588 (66.0)	63 (10.7)	525 (89.3)
Smoke exposure ^a^			
Yes	100 (11.2)	11 (11.0)	89 (89.0)
No	791 (88.8)	68 (8.6)	723 (91.4)
PM_2.5_ (µg/m^3^) ^b^	8.13 (1.10)	8.12 (1.11)	8.13 (1.10)
Temperature (C) ^b^	12.10 (4.31)	12.50 (4.16)	12.10 (4.33)

^a^ Defined as active cigarette smoking or exposure to environmental tobacco smoke for 1 h or more during pregnancy. ^b^ Average exposure across the first 32 weeks of gestation.

## Data Availability

Data are not publicly available due to human subjects confidentiality concerns; however, a minimally sufficient dataset can be obtained by reasonable request to the corresponding author.

## Supplementary Materials

The following are available online at https://www.mdpi.com/article/10.3390/toxics9120352/s1, Figure S1: Diagram of analytic sample selection, Figure S2: Directed Acyclic Graph describing assumed relationships between PM_2.5_, preterm birth, and covariates, Figure S3: Distribution of PM_2.5_ by preterm birth status, Figure S4: Posterior mean and 95% credible intervals from an intercept-only CWVS model examining an interquartile range increase in weekly PM_2.5_ in relation to the odds of preterm birth, Figure S5: Posterior mean and 95% credible intervals from adjusted CWVS models examining an interquartile range increase in weekly PM_2.5_ in relation to the odds of preterm birth stratified by spontaneous versus iatrogenic preterm birth phenotype, Figure S6: Posterior mean and 95% credible intervals from adjusted CWVS models examining an interquartile range increase in weekly PM_2.5_ in relation to the odds of preterm birth excluding participants diagnosed with gestational hypertension, pre-eclampsia/eclampsia, or gestational diabetes during pregnancy (*n* = 138) and those missing information on these conditions (*n* = 45), Figure S7: Posterior mean and 95% credible intervals from adjusted CWVS models examining an interquartile range increase in weekly PM_2.5_ in relation to the odds of preterm birth additionally adjusted for season of last menstrual period, Figure S8: Posterior mean and 95% credible intervals from adjusted CWVS models examining an interquartile range increase in weekly PM_2.5_ in relation to the odds of preterm birth additionally adjusted for year of birth, Figure S9: Posterior mean and 95% credible intervals from adjusted CWVS models examining an interquartile range increase in weekly PM_2.5_ in relation to the odds of preterm birth additionally adjusted for study site (Boston vs. New York City). Table S1: Participant characteristics for those included in the analytic sample compared to excluded from the analytic sample as described in Figure S1.

## References

[1] Martin J.A., Hamilton B.E., Osterman M.J.K., Driscoll A.K. (2021). Births: Final Data for 2019.

[2] Crump C., Sundquist J., Winkleby M.A., Sundquist K. (2019). Gestational age at birth and mortality from infancy into mid-adulthood: A national cohort study. Lancet Child Adolesc. Health.

[3] Markopoulou P., Papanikolaou E., Analytis A., Zoumakis E., Siahanidou T. (2019). Preterm Birth as a Risk Factor for Metabolic Syndrome and Cardiovascular Disease in Adult Life: A Systematic Review and Meta-Analysis. J. Pediatr..

[4] Moster D., Lie R.T., Markestad T. (2008). Long-term medical and social consequences of preterm birth. N. Engl. J. Med..

[5] Petrou S., Eddama O., Mangham-Jefferies L. (2010). A structured review of the recent literature on the economic consequences of preterm birth. Arch. Dis. Child.-Fetal Neonatal Ed..

[6] Klepac P., Locatelli I., Korošec S., Künzli N., Kukec A. (2018). Ambient air pollution and pregnancy outcomes: A comprehensive review and identification of environmental public health challenges. Environ. Res..

[7] Stieb D.M., Chen L., Eshoul M., Judek S. (2012). Ambient air pollution, birth weight and preterm birth: A systematic review and meta-analysis. Environ. Res..

[8] Wilson A., Chiu Y.-H.M., Hsu H.-H.L., Wright R., Wright R.J., Coull B.A. (2017). Bayesian distributed lag interaction models to identify perinatal windows of vulnerability in children’s health. Biostatistics.

[9] Wright R. (2017). Environment, susceptibility windows, development, and child health. Curr. Opin. Pediatr..

[10] Chang H.H., Warren J.L., Darrow L.A., Reich B.J., Waller L.A. (2015). Assessment of critical exposure and outcome windows in time-to-event analysis with application to air pollution and preterm birth study. Biostatistics.

[11] Wang Q., Benmarhnia T., Zhang H., Knibbs L.D., Sheridan P., Li C., Bao J., Ren M., Wang S., He Y. (2018). Identifying windows of susceptibility for maternal exposure to ambient air pollution and preterm birth. Environ. Int..

[12] Warren J., Fuentes M., Herring A., Langlois P. (2012). Spatial-Temporal Modeling of the Association between Air Pollution Exposure and Preterm Birth: Identifying Critical Windows of Exposure. Biometrics.

[13] Chu C., Zhu Y., Liu C., Chen R., Yan Y., Ren Y., Li X., Wang J., Ge W., Kan H. (2021). Ambient fine particulate matter air pollution and the risk of preterm birth: A multicenter birth cohort study in China. Environ. Pollut..

[14] Liu Y., Xu J., Chen D., Sun P., Ma X. (2019). The association between air pollution and preterm birth and low birth weight in Guangdong, China. BMC Public Health.

[15] Inkster A., Fernández-Boyano I., Robinson W. (2021). Sex Differences Are Here to Stay: Relevance to Prenatal Care. J. Clin. Med..

[16] Ghosh R., Rankin J., Pless-Mulloli T., Glinianaia S. (2007). Does the effect of air pollution on pregnancy outcomes differ by gender? A systematic review. Environ. Res..

[17] Brunst K.J., Sanchez-Guerra M., Chiu Y.-H.M., Wilson A., Coull B.A., Kloog I., Schwartz J., Brennan K.J., Enlow M.B., Wright R.O. (2018). Prenatal particulate matter exposure and mitochondrial dysfunction at the maternal-fetal interface: Effect modification by maternal lifetime trauma and child sex. Environ. Int..

[18] Just A.C., Arfer K.B., Rush J., Dorman M., Shtein A., Lyapustin A., Kloog I. (2020). Advancing methodologies for applying machine learning and evaluating spatiotemporal models of fine particulate matter (PM2.5) using satellite data over large regions. Atmos. Environ..

[19] Carrión D., Arfer K.B., Rush J., Dorman M., Rowland S.T., Kioumourtzoglou M.-A., Kloog I., Just A.C. (2021). A 1-km hourly air-temperature model for 13 northeastern U.S. states using remotely sensed and ground-based measurements. Environ. Res..

[20] Ada M.L., Hacker M.R., Golen T.H., Haviland M., Shainker S.A., Burris H.H. (2017). Trends in provider-initiated versus spontaneous preterm deliveries, 2004–2013. J. Perinatol..

[21] Textor J., van der Zander B., Gilthorpe M.S., Liśkiewicz M., Ellison G.T. (2017). Robust causal inference using directed acyclic graphs: The R package ‘dagitty’. Int. J. Epidemiol..

[22] Kloog I. (2019). Air pollution, ambient temperature, green space and preterm birth. Curr. Opin. Pediatr..

[23] Warren J.L., Kong W., Luben T., Chang H.H. (2019). Critical window variable selection: Estimating the impact of air pollution on very preterm birth. Biostatistics.

[24] Gehring U., Wijga A.H., Fischer P., de Jongste J.C., Kerkhof M., Koppelman G.H., Smit H.A., Brunekreef B. (2011). Traffic-related air pollution, preterm birth and term birth weight in the PIAMA birth cohort study. Environ. Res..

[25] Hannam K., McNamee R., Baker P., Sibley C., Agius R. (2014). Air pollution exposure and adverse pregnancy outcomes in a large UK birth cohort: Use of a novel spatio-temporal modelling technique. Scand. J. Work. Environ. Health.

[26] Lavigne E., Yasseen A.S., Stieb D.M., Hystad P., van Donkelaar A., Martin R., Brook J.R., Crouse D., Burnett R.T., Chen H. (2016). Ambient air pollution and adverse birth outcomes: Differences by maternal comorbidities. Environ. Res..

[27] Pereira G., Bell M.L., Lee H.J., Koutrakis P., Belanger K. (2014). Sources of Fine Particulate Matter and Risk of Preterm Birth in Connecticut, 2000–2006: A Longitudinal Study. Environ. Health Perspect..

[28] Qian Z., Liang S., Yang S., Trevathan E., Huang Z., Yang R., Wang J., Hu K., Zhang Y., Vaughn M. (2016). Ambient air pollution and preterm birth: A prospective birth cohort study in Wuhan, China. Int. J. Hyg. Environ. Health.

[29] Wu J., Ren C., Delfino R.J., Chung J., Wilhelm M., Ritz B. (2009). Association between Local Traffic-Generated Air Pollution and Preeclampsia and Preterm Delivery in the South Coast Air Basin of California. Environ. Health Perspect..

[30] Tanner J.P., Salemi J.L., Stuart A.L., Yu H., Jordan M.M., Duclos C., Cavicchia P., Correia J.A., Watkins S.M., Kirby R.S. (2016). Uncertainty in maternal exposures to ambient PM2.5 and benzene during pregnancy: Sensitivity to exposure estimation decisions. Spat. Spatio-Temporal Epidemiol..

[31] Rappazzo K.M., Daniels J.L., Messer L.C., Poole C., Lobdell D. (2014). Exposure to Fine Particulate Matter during Pregnancy and Risk of Preterm Birth among Women in New Jersey, Ohio, and Pennsylvania, 2000–2005. Environ. Health Perspect..

[32] Symanski E., Davila M., McHugh M.K., Waller D.K., Zhang X., Lai D. (2014). Maternal Exposure to Fine Particulate Pollution During Narrow Gestational Periods and Newborn Health in Harris County, Texas. Matern. Child Health J..

[33] Yuan L., Zhang Y., Wang W., Chen R., Liu Y., Liu C., Kan H., Gao Y., Tian Y. (2020). Critical windows for maternal fine particulate matter exposure and adverse birth outcomes: The Shanghai birth cohort study. Chemosphere.

[34] Sheridan P., Ilango S., Bruckner T.A., Wang Q., Basu R., Benmarhnia T. (2019). Ambient Fine Particulate Matter and Preterm Birth in California: Identification of Critical Exposure Windows. Am. J. Epidemiol..

[35] Altman M.R., Baer R.J., Jelliffe-Pawlowski L.L. (2018). Patterns of Preterm Birth among Women of Native Hawaiian and Pacific Islander Descent. Am. J. Perinatol..

[36] Darrow L.A., Klein M., Flanders W.D., Waller L.A., Correa A., Marcus M., Mulholland J.A., Russell A.G., Tolbert P.E. (2009). Ambient air pollution and preterm birth: A time-series analysis. Epidemiology.

[37] Jalaludin B., Mannes T., Morgan G., Lincoln D., Sheppeard V., Corbett S. (2007). Impact of ambient air pollution on gestational age is modified by season in Sydney, Australia. Environ. Health.

[38] Wilhelm M., Ghosh J.K., Su J., Cockburn M., Jerrett M., Ritz B. (2011). Traffic-related air toxics and preterm birth: A population-based case-control study in Los Angeles county, California. Environ. Health.

[39] Wilhelm M., Ritz B. (2005). Local Variations in CO and Particulate Air Pollution and Adverse Birth Outcomes in Los Angeles County, California, USA. Environ. Health Perspect..

[40] Blum J.L., Chen L.-C., Zelikoff J.T. (2017). Exposure to Ambient Particulate Matter during Specific Gestational Periods Produces Adverse Obstetric Consequences in Mice. Environ. Health Perspect..

[41] Veras M.M., Damaceno-Rodrigues N.R., Caldini E., Ribeiro A.A.C.M., Mayhew T.M., Saldiva P., Dolhnikoff M. (2008). Particulate Urban Air Pollution Affects the Functional Morphology of Mouse Placenta1. Biol. Reprod..

[42] Nääv Å., Erlandsson L., Isaxon C., Frostner E.Å., Ehinger J., Sporre M., Krais A.M., Strandberg B., Lundh T., Elmér E. (2020). Urban PM2.5 Induces Cellular Toxicity, Hormone Dysregulation, Oxidative Damage, Inflammation, and Mitochondrial Interference in the HRT8 Trophoblast Cell Line. Front. Endocrinol..

[43] Hsiao E.Y., Patterson P.H. (2011). Activation of the maternal immune system induces endocrine changes in the placenta via IL-6. Brain Behav. Immun..

[44] Keenan-Devlin L.S., Caplan M., Freedman A., Kuchta K., Grobman W., Buss C., Adam E.K., Entringer S., Miller G.E., Borders A.E.B. (2021). Using principle component analysis to examine associations of early pregnancy inflammatory biomarker profiles and adverse birth outcomes. Am. J. Reprod. Immunol..

[45] Braun D.A., Ishii Y., Walsh A., Van Allen E.M., Wu C.J., Shukla S.A., Choueiri T.K. (2019). Clinical Validation of PBRM1 Alterations as a Marker of Immune Checkpoint Inhibitor Response in Renal Cell Carcinoma. JAMA Oncol..

