# Supplementary material for: Spatially and Temporally Resolved Ambient PM2.5 in Relation to Preterm Birth
Source: Toxics. 2021 Dec 14;9(12):352. doi: 10.3390/toxics9120352 (PMC8708619; doi:10.3390/toxics9120352)
Supplement: Supplementary file 1 [file toxics-09-00352-s001.zip › toxics-1450521-supplementary.pdf]

toxics MDPI

Supplementary Materials: Spatially and Temporally Resolved Ambient $PM_{2.5}$ in Relation to Preterm Birth

Whitney Cowell, Elena Colicino, Xueying Zhang, Rachel Ledyard, Heather H. Burris, Michele R. Hacker, Itai Kloog, Allan Just, Robert O. Wright and Rosalind J. Wright

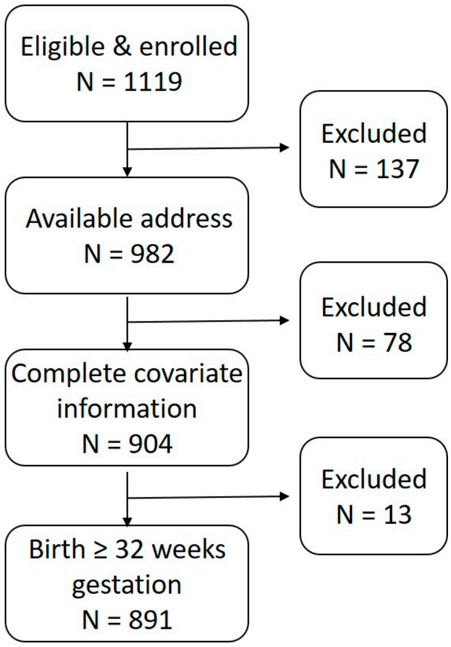

Figure S1. Diagram of analytic sample selection.

Table S1. Participant characteristics for those included in the analytic sample compared to those excluded from the analytic sample as described in Figure S1.

	Included (N = 891)		Excluded (N = 228)	
	N (%) or mean±SD	N	N (%) or mean±SD	N
Maternal age	29.1 ± 5.8	891	28.2 ± 6.0	228
Race/ethnicity		891		129
White, non-Hispanic	147 (16.5)		6 (4.7)	
White-Hispanic	317 (35.6)		54 (41.9)	
Black/Black-Hispanic	385 (43.2)		67 (51.9)	
Other	42 (4.7)		2 (1.6)	
Education		891		117
<High school	183 (20.5)		14 (12.0)	
High school degree	444 (49.8)		86 (73.5)	
>High school	264 (29.6)		17 (14.5)	
Nulliparous	303 (34.0)	891	59 (33.7)	175
Smoke exposure	100 (11.2)	891	32 (23.7)	135

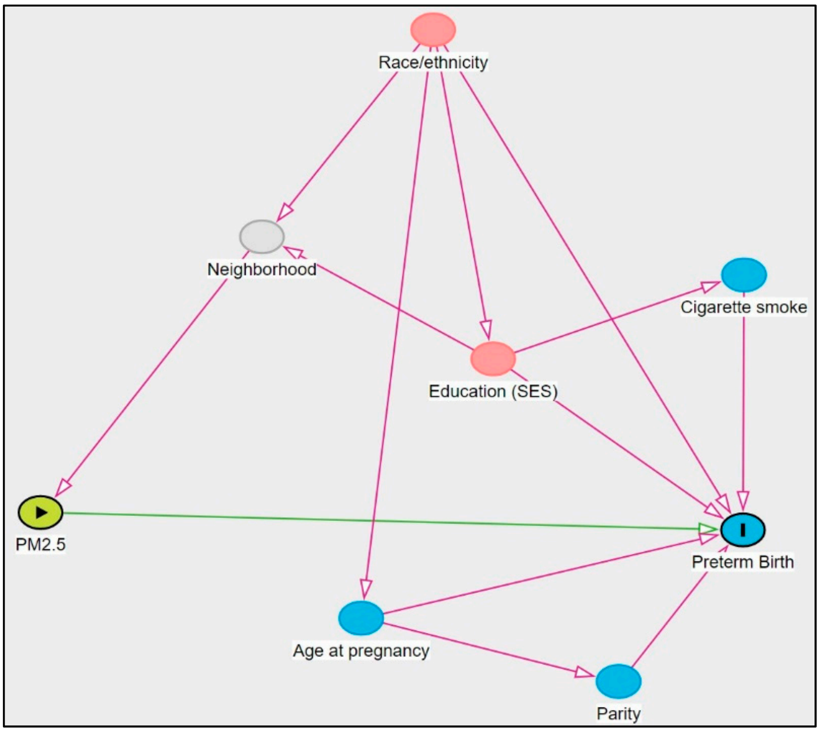

Figure S2. Directed Acyclic Graph of assumed conditional dependencies between the exposure, outcome, and covariates.

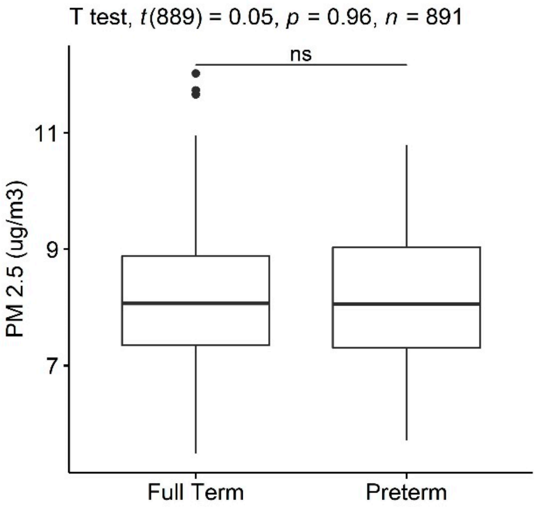

Figure S3. Distribution of $PM_{2.5}$ by preterm birth status. ns = non-significant.

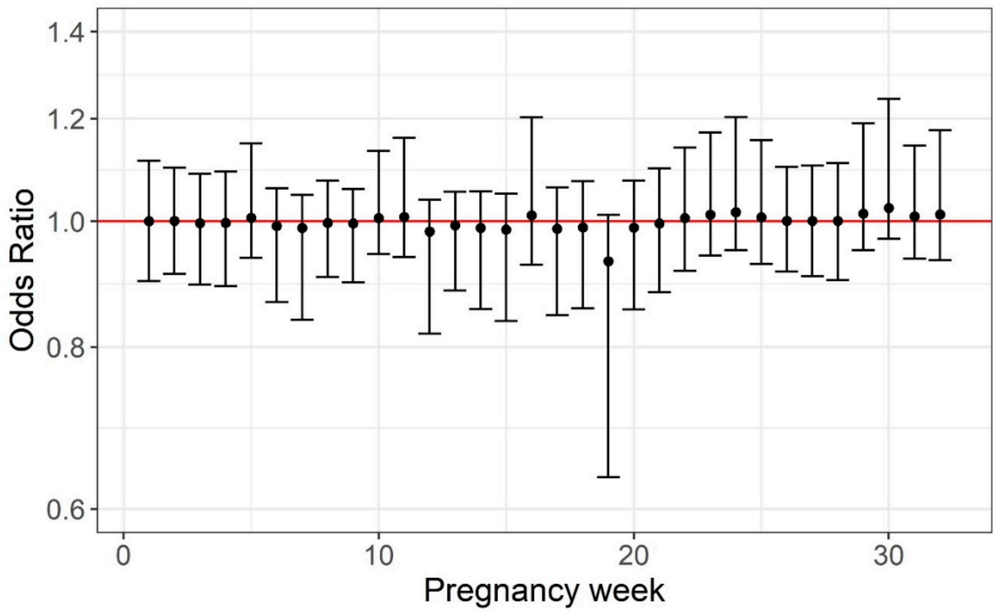

Figure S4. Posterior mean and 95% credible intervals from an intercept-only CWVS model examining an interquartile range increase in weekly $PM_{2.5}$ in relation to the odds of preterm birth.

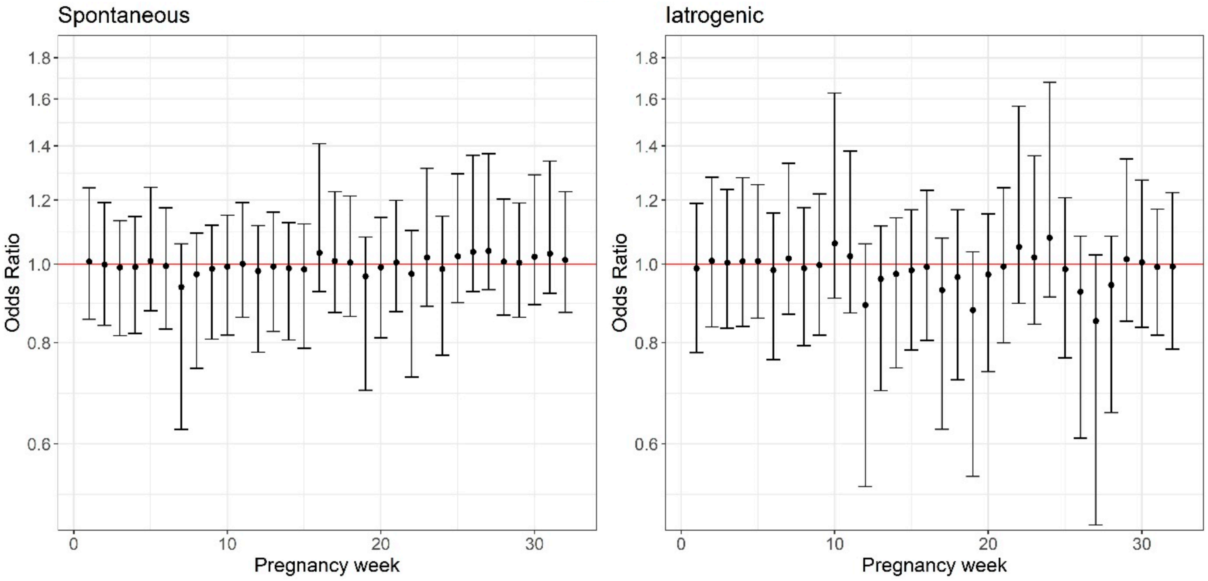

Figure S5. Posterior mean and 95% credible intervals from adjusted CWVS models examining an interquartile range increase in weekly $PM_{2.5}$ in relation to the odds of preterm birth stratified by spontaneous versus iatrogenic preterm birth phenotype.

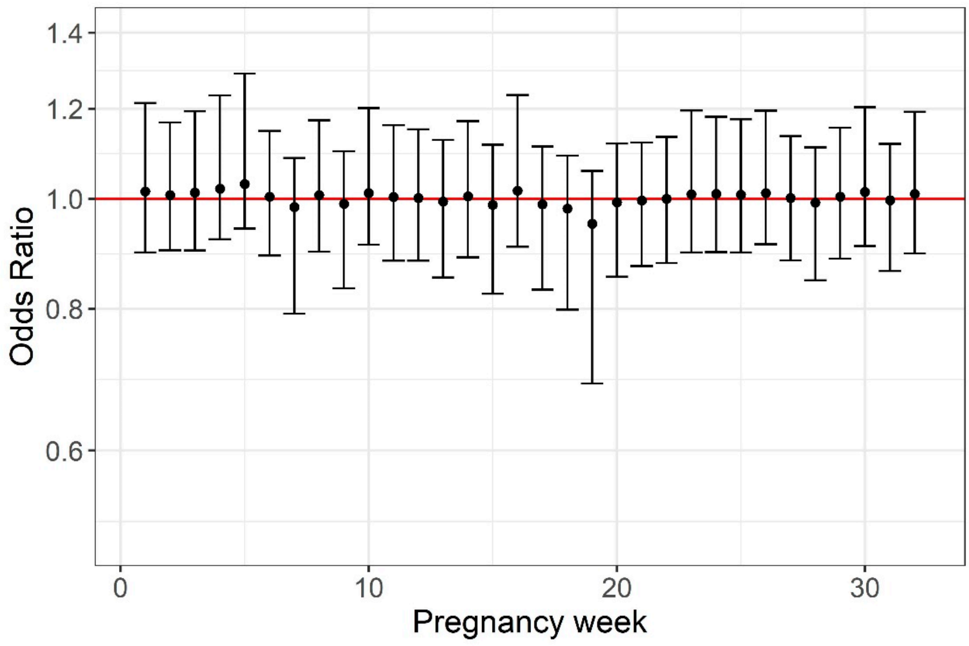

Figure S6. Posterior mean and 95% credible intervals from adjusted CWVS models examining an interquartile range increase in weekly $PM_{2.5}$ in relation to the odds of preterm birth excluding participants diagnosed with gestational hypertension, pre-eclampsia/eclampsia, or gestational diabetes during pregnancy (N = 138) and those missing information on these conditions (N = 45).

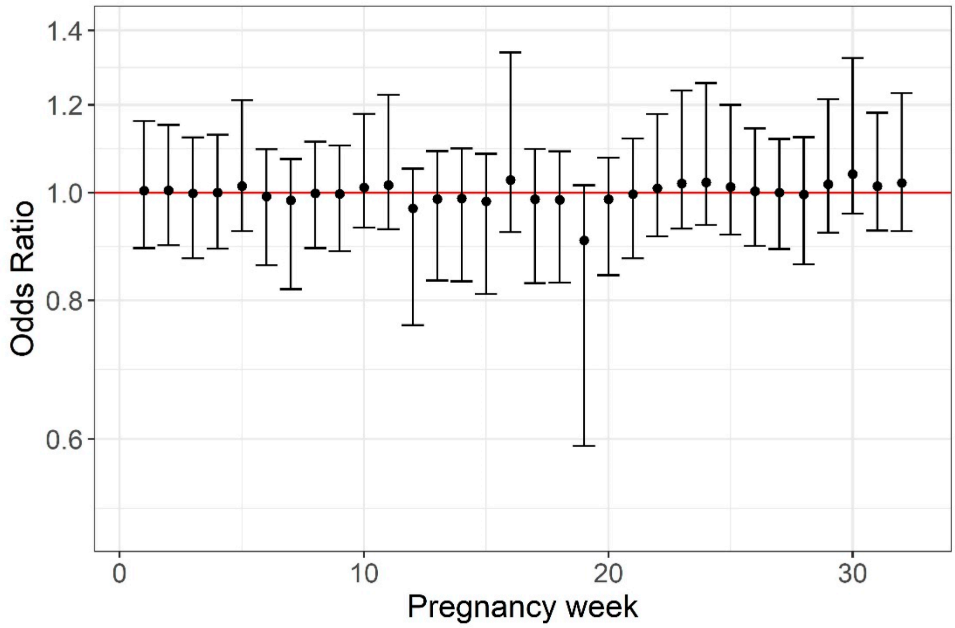

Figure S7. Posterior mean and 95% credible intervals from adjusted CWVS models examining an interquartile range increase in weekly $PM_{2.5}$ in relation to the odds of preterm birth additionally adjusted for season of last menstrual period.

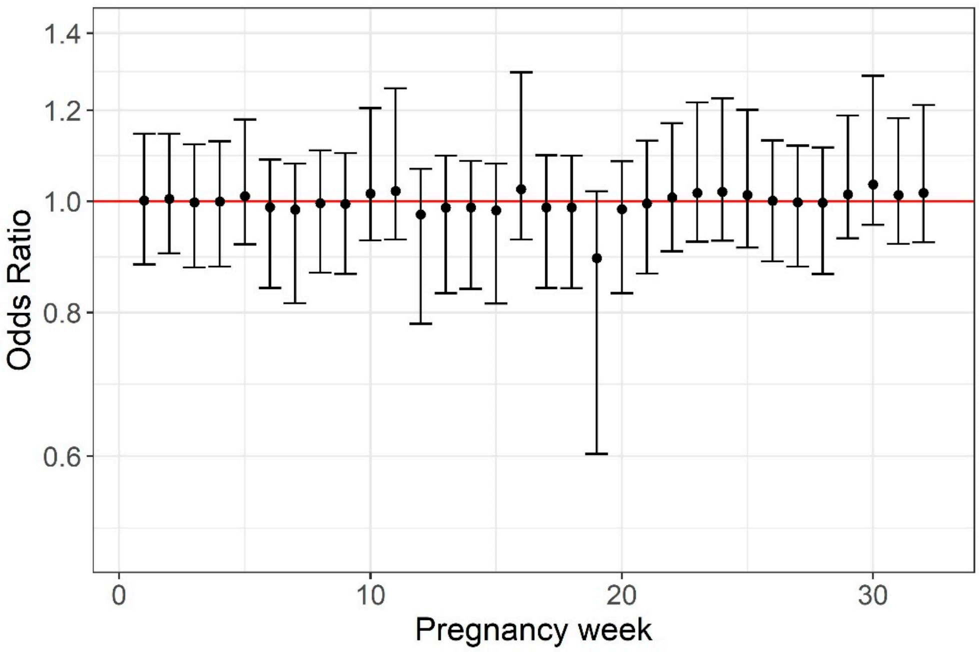

Figure S8. Posterior mean and 95% credible intervals from adjusted CWVS models examining an interquartile range increase in weekly $PM_{2.5}$ in relation to the odds of preterm birth additionally adjusted for year of birth.

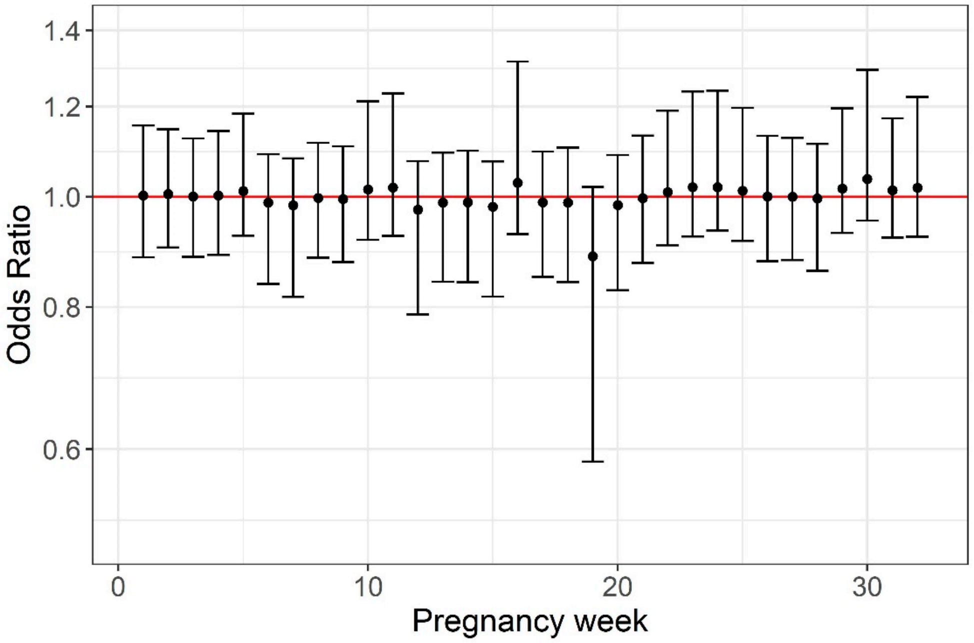

Figure S9. Posterior mean and 95% credible intervals from adjusted CWVS models examining an interquartile range increase in weekly $PM_{2.5}$ in relation to the odds of preterm birth additionally adjusted for study site (Boston vs. New York City).
